# Mesocorticolimbic circuit mechanisms of social dominance behavior

**DOI:** 10.1038/s12276-024-01299-8

**Published:** 2024-09-02

**Authors:** Tae-Yong Choi, Sejin Jeong, Ja Wook Koo

**Affiliations:** 1 Emotion, Cognition and Behavior Research Group, Korea Brain Research Institute, Daegu, Republic of Korea; 2 Department of Life Sciences, Yeungnam University, Gyeongsan, Republic of Korea; 3 Department of Brain Sciences, Daegu Gyeongbuk Institute of Science and Technology, Daegu, Republic of Korea

**Keywords:** Social neuroscience, Reward, Motivation, Emotion

## Abstract

Social animals, including rodents, primates, and humans, partake in competition for finite resources, thereby establishing social hierarchies wherein an individual’s social standing influences diverse behaviors. Understanding the neurobiological underpinnings of social dominance is imperative, given its ramifications for health, survival, and reproduction. Social dominance behavior comprises several facets, including social recognition, social decision-making, and actions, indicating the concerted involvement of multiple brain regions in orchestrating this behavior. While extensive research has been dedicated to elucidating the neurobiology of social interaction, recent studies have increasingly delved into adverse social behaviors such as social competition and hierarchy. This review focuses on the latest advancements in comprehending the mechanisms of the mesocorticolimbic circuit governing social dominance, with a specific focus on rodent studies, elucidating the intricate dynamics of social hierarchies and their implications for individual well-being and adaptation.

## Understanding social hierarchies: rodent studies shed light on brain mechanisms

In the animal world, the fight for food and mates often results in social rankings, with dominant animals getting better access to resources. This review explores the brain biology of social dominance, focusing on specific brain circuits in rodents. Using behavioral tests, they’ve started to understand how different brain areas and their connections affect social ranking among animals. The study combines results from many experiments to better understand how social dominance is wired in the brain. The results highlight the complexity of social dominance, showing it as a trait influenced by multiple brain areas and their interactions. They conclude that understanding these brain processes is key for understanding the wider implications of social behavior in health and disease. Their work improves our understanding of the biological basis of social hierarchies, suggesting potential targets for treating social behavior disorders.

This summary was initially drafted using artificial intelligence, then revised and fact-checked by the author.

## Introduction

Social animals, including rodents, primates, and humans, compete to obtain scarce resources. This social competition gives rise to a social hierarchy within a group, where an individual’s social status influences various behaviors. For example, social dominants readily secure sufficient food and sexual partners, whereas social subordinates tend to avoid competition with social dominants^1,2^. These diverse behavioral adaptations play pivotal roles in bolstering individuals’ health, survival, and reproduction^3,4^. Therefore, understanding the neurobiological mechanisms underpinning social dominance behavior is crucial.

Social dominance behavior involves multiple processes. Animals recognize each other within the context of social interaction (i.e., social recognition)^5,6^. In particular, animals discern the social position of their counterparts and then assess whether to engage in favorable interactions or to compete with others (i.e., social decision-making)^7,8^. When animals are familiar with one another, social interactions typically occur without aggression. However, if one animal holds a higher social rank than another, the dominant individual may assert dominance through aggression, while subordinates tend to avoid confrontation with dominant individuals. This entire process encompasses social recognition, social decision-making, and subsequent actions, indicating the involvement of various brain regions in orchestrating social dominance behavior.

The neurobiological mechanisms underlying favorable social interaction behavior, commonly referred to as sociability or social preference, have been extensively studied^9–11^. Both human patients and animal models of many neuropsychiatric disorders, such as autism spectrum disorder and depression, often exhibit deficits in sociability^12–20^. Numerous brain regions, including the medial prefrontal cortex (mPFC), nucleus accumbens (NAc), and ventral tegmental area (VTA), which are key components of the mesocorticolimbic circuit, play crucial roles in mediating this behavior^21–29^. Conversely, research into unfavorable social behaviors, such as social competition and dominance behavior, has been increasingly pursued in recent years.

Here, we provide a comprehensive review of the mesocorticolimbic and corticostriatal circuit mechanisms underlying social dominance behavior, with a particular focus on the latest advancements in animal studies, notably those involving rodents. Furthermore, we outline the various behavioral methodologies employed to investigate social dominance behavior and its behavioral and pathological correlates. Finally, we critically evaluate the limitations of prior research elucidating the mechanisms of social dominance behavior and propose potential future research directions.

## How to study social dominance behavior using rodents

### Tube test

**Fig. 1:**
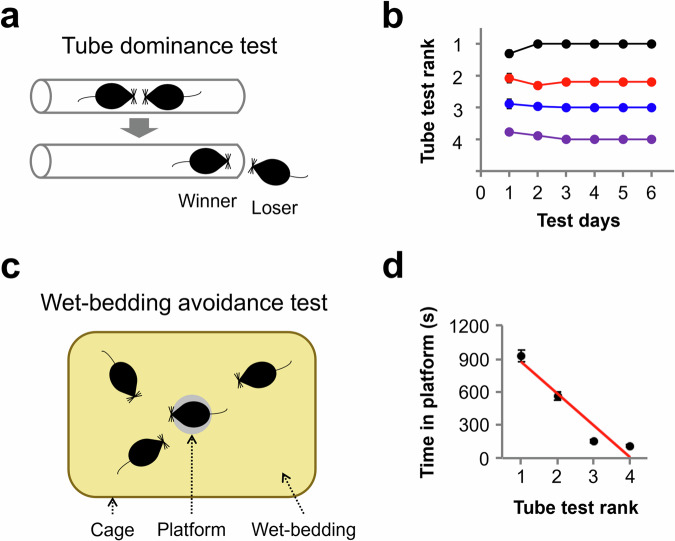
The tube test and wet bedding avoidance (WBA) test were used to study social dominance behavior in rodents. **a** Schematic illustration of the tube test. **b** Summary graph for 22 cages of 4 mice with stable ranks measured daily over 6 days. **c** Schematic illustration of the WBA test. Four cagemate mice competed for an elevated platform in the center of a cage to avoid wet bedding. **d** Correlation between the amount of time spent on the platform and the rank in the tube test. The images in (**c**, **d**) are adapted from Choi et al. with permission^34^.

The tube test was originally developed in the 1960s to compare social dominance among mice of different strains^30^. This test involves placing two animals in a narrow tube with enough space for only one animal to pass through at a time. This approach allows observation of competitive behavior in rodents as they encounter each other within the narrow tube, elucidating the formation of social hierarchy. Various behaviors exhibited during the tube test are interpreted as actions aimed at securing limited resources or territory. For example, push behavior involves an individual’s natural movement forward, pushing the other individual to occupy the space previously inhabited by it. Conversely, retreat behavior, although less instinctive, involves moving backward, either passively (in response to the other individual’s push) or voluntarily (without external pressure), thereby yielding space to the other individual. Through this test, researchers have gained insights into the social hierarchy and dominant behaviors of rodents^31–33^. The tube test is commonly employed in studies of social dominance behavior due to its simple equipment and procedure, as well as its straightforward and intuitive results (Fig. 1a, b). Additionally, social ranks determined through the tube test have demonstrated a high correlation with the results of other behavioral experiments introduced below, which are used to assess social hierarchy^31^.

### Wet bedding avoidance test

To determine whether the results of the tube test can be extrapolated to other social dominance behaviors, a recent study introduced a new behavioral assay called the wet bedding avoidance (WBA) test. This test assesses individuals’ territorial instincts when occupying a platform to avoid wet bedding in a cage. In the WBA test, social rank is established by evaluating the degree of platform occupancy (Fig. 1c). The social hierarchy identified through the WBA test showed a strong correlation with that determined by the tube test (Fig. 1d). Furthermore, changes in social rank observed in the tube test due to the regulation of gene expression were similarly detected in the WBA test^34^.

### Warm spot test

The warm spot test is a behavioral test developed by Zhou and colleagues that leverages animals’ innate tendency to occupy more favorable areas to assess social dominance^35^. In this test, multiple cagemate mice are initially placed in a cage with a floor temperature of 0 °C for 30 min. Subsequently, they are transferred to a cage with the same floor temperature of 0 °C but with a small spot in one corner where the temperature is maintained at 34 °C. The degree to which they occupy the warm spot differs according to their social hierarchy. The warm spot test results are strongly correlated with those of the tube test^35–37^.

### Urine marking assay

Urine marking is a common behavior in many rodent species and involves the deposition of small amounts of urine in specific areas or on objects within their territory. Rodents use urine marking to communicate with other members of their species, establish territory boundaries, and convey information about their reproductive status. In general, social dominants urinate in a broader area than social subordinates in a cage. The urine marking assay is valuable for researchers interested in understanding various aspects of rodent behavior, including territoriality, social communication, and the influence of hormones or environmental factors on urine marking patterns. These findings can provide insights into the social dynamics and reproductive strategies of rodents, as well as their responses to changes in their surroundings^38–40^.

### Reward competition test

The reward competition test in rodents is a behavioral experiment designed to study how rodents compete for limited resources (e.g., food and water) within a controlled environment. This test is often used in research to investigate social hierarchies, dominance behaviors, and competition dynamics among rodents, such as mice or rats. Reward consumption is not only essential for survival but also one of the most potent motivating factors. Rodents, typically deprived of food or water, are placed in an enclosure or cage with restricted access to these resources, such as a single source or a limited quantity. Researchers closely observe the rodents’ interactions around the reward resource, recording behaviors such as aggressive interactions, dominance displays, or attempts to access the reward. Reward competition tests can provide valuable information about the social dynamics of rodents, including how they establish hierarchies and resolve conflicts over limited resources such as food or water. Normal chow is typically used in this test, but it can also be replaced with normal water, palatable food, or liquid^41–43^. Cordero and Sandi notably used food as the competitive resource for establishing a social hierarchy during the initial test on Day 1 and water as an additional competitive resource to evaluate the long-term establishment of a social hierarchy during the subsequent test on Day 8. They stated that the primary rationale underlying transitioning the reward from food to water in the competition test was to forestall the re-establishment of a social hierarchy during the second test based on memory of the test and/or reward modality. This decision was made with the specific aim of assessing the subject’s memory^44^.

### Agonistic behavior

Agonistic behavior in rodents refers to a set of behaviors related to conflict, aggression, and social dominance within their social groups or territories. These behaviors can be observed when mice or rats interact with each other in competitive situations, reflecting social hierarchies. Agonistic behavior includes various actions and postures and can be categorized into both offensive and defensive behaviors^45–47^. Some of the key reasons why rodents display agonistic behavior are as follows:

1. Resource competition: Mice often engage in agonistic behavior when they compete for limited resources such as food, water, nesting sites, and territory. Aggressive interactions can help individuals gain access to these essential resources.
2. Territorial defense: Mice can be territorial animals, and agonistic behavior may be exhibited when defending their established territory against intruders from other social groups.
3. Reproductive competition: In some cases, agonistic behavior can also be related to reproductive competition. Male mice, for example, may engage in aggressive interactions to gain access to females during the breeding season.
4. Establishment of social hierarchy: Agonistic behavior is a means through which mice establish and maintain a social hierarchy within their groups. Dominant-subordinate relationships help reduce the frequency of physical conflicts, as individuals recognize their rank in the hierarchy.

It is important to note that not all mouse interactions are aggressive, and mice also engage in nonagonistic social behaviors, such as grooming and social bonding. Agonistic behavior serves specific functions within social structure and ecology, helping to regulate competition and maintain group order.

### Visible burrow system

The visible burrow system (VBS) is an experimental setup used in ecological and behavioral research for studying the behavior and social dynamics of burrowing animals. The VBS is designed to simulate a burrowing environment in a laboratory setting, enabling researchers to observe and record the activities of burrowing animals^48–50^. The VBS comprises transparent pipes and tunnels, allowing real-time observation of mouse activities within burrows. To determine social hierarchy, researchers observe the behaviors of mice within the burrow, identifying the mice that are more frequently or higher positioned than others. In addition, researchers observe and record feeding behavior to analyze food competition and behavioral patterns between socially dominant and subordinate mice. Dominant mice often access food first and may chase other mice away.

### Barbering behavior

In group-living mice, it is common to find individuals with their whiskers or fur removed, which is considered a type of social dominance behavior where dominant individuals remove the fur or whiskers of subordinate individuals, also known as the Dalila effect. In general, barbering is a kind of agonistic behavior that is typically believed to occur only in laboratory mice and not in naturalistic environments^51–53^.

## Brain regions and circuits related to social dominance behavior

### Medial prefrontal cortex

Human brain imaging studies have revealed that the medial prefrontal cortex (mPFC) plays a significant role in social dominance behavior^54,55^. However, the detailed mechanisms by which the mPFC is involved in this behavior are being elucidated through various rodent studies.

**Fig. 2:**
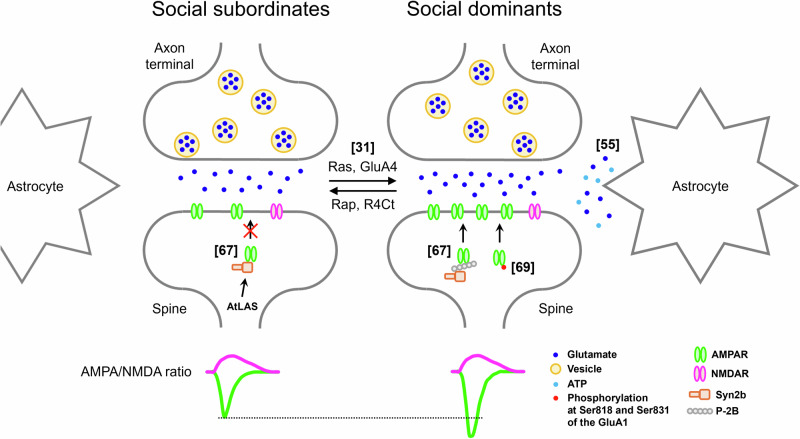
Synaptic efficacy in the mPFC bidirectionally regulates social hierarchy. Excitatory synaptic transmission in the mPFC is greater in social dominants than in subordinates. Synaptic potentiation induced by the expression of Ras or GluA4 in the mPFC increases social hierarchy, whereas social rank is decreased by the expression of Rap or R4Ct in the mPFC^31^. Glutamate and ATP released from astrocytes in the mPFC regulate synaptic efficacy, resulting in changes in social hierarchy^55^. Antisense long noncoding RNA of synapsin II (AtLAS) regulates the expression of synapsin 2b (Syn2b), which binds to AMPA receptors (AMPARs) to inhibit the delivery of AMPARs to synapses, resulting in a reduction in social rank. However, the delivery of AMPARs to synapses by the expression of a membrane-permeable peptide (P-2B) via the fusion of the TAT sequence to the C-terminus of Syn2b disrupts the binding of Syn2b to AMPARs, resulting in an increase in social status^67^. AMPAR trafficking to the synapse by the phosphorylation of Ser818 and Ser831 of the GluA1 subunit is highly correlated with social winning in the tube test^69^.

**Fig. 3:**
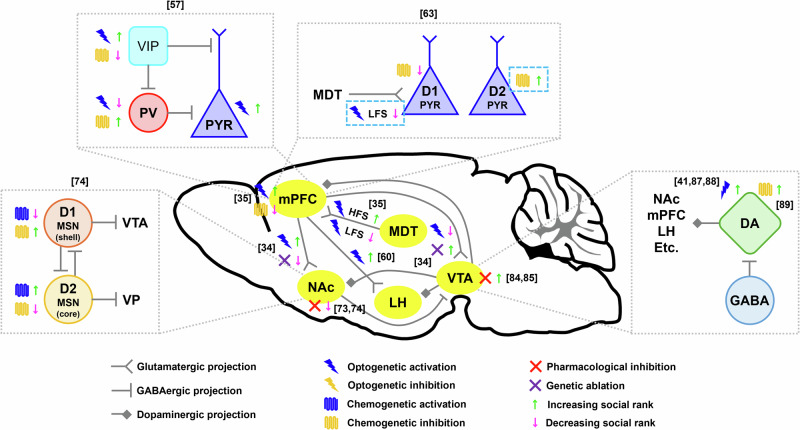
Regulation of social dominance behavior by manipulating mesocorticolimbic circuit activities. Optogenetic activation or chemogenetic inhibition of the mPFC increases or decreases social dominance, respectively. Synaptic potentiation-inducing high-frequency stimulation (HFS) at inputs from the mediodorsal thalamus (MDT) to the mPFC increases social ranks, whereas synaptic depression-inducing low-frequency stimulation (LFS) on MDT-mPFC projection decreases social ranks^35^. The disinhibitory VIP–PV–PYR microcircuit in the mPFC computes information related to social hierarchy^57^. Prefrontal D1- and D2-expressing neurons distinctly regulate social dominance behavior^63^. Optogenetic activation of mPFC neurons projecting to the LH increases winnings during the food competition test^60^. mPFC neuronal populations that project to the NAc or VTA conversely regulate social dominance^34^. Pharmacological inhibition of the NAc decreases social dominance^73,74^. In contrast, D1- and D2-expressing neurons in the NAc regulate social hierarchy^74^. Pharmacological inhibition of the VTA increases social hierarchy^84,85^. Optogenetic activation of VTA neurons expressing dopamine (DA) decreases reward latency during the reward competition test^41^ and increases agonistic and aggressive behaviors^87^.

Previous studies revealed that synaptic efficacy in the mPFC, especially in the prelimbic cortex (PrL) or dorsomedial PFC (dmPFC), is the main contributor to social dominance. Social dominants exhibited greater amplitudes of miniature excitatory synaptic currents (mEPSCs) in the mPFC than subordinates (Fig. 2)^31,56^. In addition, enhancing synaptic efficacy in the mPFC of social subordinates through the expression of Ras or GluR4 led to an increase in social rank, while reducing synaptic efficacy in the mPFC of social dominants through the expression of Rap or R4Ct resulted in a reduction in social rank (Fig. 2)^31^. High-frequency stimulation (HFS), which induces long-term potentiation (LTP), or low-frequency stimulation (LFS), which induces long-term depression (LTD), in axon terminals from the mediodorsal thalamus (MDT) to the mPFC results in an elevation or reduction of social hierarchy (Fig. 3)^35^.

Social hierarchy is also affected by neuronal activity in the mPFC. The number of activated neurons that expressed c-fos^31^, intrinsic excitability^56^, and firing rates^37^ in the mPFC were greater in social dominants than in subordinates. Studies using in vivo single-unit recording or calcium imaging revealed that effortful behaviors to win during the tube test, such as pushing, increase the activity of mPFC excitatory neurons^35,57–59^. In addition, neuronal activity in the mPFC was increased when a mouse guarded or extorted food pellets during the food competition test^43^. Recent studies using the food competition test and wireless in vivo recordings revealed that distinct neuronal ensembles in the mPFC or anterior cingulate cortex (ACC) encode an animal’s relative social rank and predict the results of competition^60,61^. Modifying neuronal activity in the mPFC using optogenetic activation or chemogenetic inhibition resulted in an increase or decrease in social rank, respectively (Fig. 3)^35,56,58,60,61^.

The balance between excitation and inhibition (E/I balance) in the neocortex is critical for various behaviors. Notably, the E/I balance is regulated by neuronal activity and synaptic transmission. These findings suggest that the E/I balance in the mPFC may affect social dominance behavior. Tan et al. reported that the deletion of tropomyosin receptor kinase B (TrkB), which recognizes brain-derived neurotrophic factor (BDNF), in corticolimbic GABAergic interneurons results in an increase in social dominance through decreasing inhibition in the mPFC. Neuronal excitability and neurite arborization were decreased in TrkB-deleted mPFC interneurons. In addition, TrkB ablation in corticolimbic GABAergic interneurons decreased the inhibitory synaptic transmission of mPFC excitatory neurons. These changes induced hyperactivation of the mPFC, resulting in a high social ranking^62^. Another study reported that both excitatory synaptic transmission in the mPFC and social dominance are increased in mutant mice lacking KCC2b, a type of K-Cl transporter KCC2 that is required for the development of the GABA switch, which causes neuronal responses to GABAergic transmission from excitatory to inhibitory early in brain development^63^. Recently, Noh et al. reported that astrocytes in the mPFC controlled social dominance behavior by regulating E/I balance. The authors found that pushing and resistance behaviors during the tube test increase not only neuronal but also astrocytic activity in the mPFC and that social dominants exhibit greater mPFC astrocytic activity than subordinates. The manipulation of mPFC astrocytes bidirectionally regulated the release of glutamate but not GABA by regulating the gliotransmission of glutamate and ATP, leading to a change in the E/I balance (Fig. 2)^56^.

Neurons in the mPFC are molecularly or anatomically heterogeneous. In addition, not only behavioral efforts to win, such as pushing but also the approach and retreat during the tube test were encoded in separate mPFC neurons^57,59^. These findings suggest that distinct neuronal subpopulations in the mPFC encode distinct social competition behaviors. Zhang et al. reported that the optogenetic activation of excitatory (or pyramidal [PYR]) neurons or vasoactive intestinal peptide (VIP)-expressing interneurons in the mPFC increases social ranks, whereas the optogenetic activation of parvalbumin (PV)-expressing interneurons in the mPFC decreases social ranks. Conversely, the chemogenetic inhibition of PV or VIP interneurons increased or decreased social hierarchy, respectively. Pushing (or retreating) increased (or decreased) mPFC activity. Before the onset of pushing behavior, VIP interneurons are activated first, followed by the activation of PYR and PV interneurons. In contrast, before the onset of retreat behavior, the activity of VIP interneurons decreased first, followed by a reduction in the activity of PYR and PV interneurons. Taken together, these findings indicate that a disinhibitory VIP-PV-PYR microcircuit in the mPFC controls social dominance behavior (Fig. 3)^58^. Another study conducted by Xing et al. reported that prefrontal neurons expressing the dopamine receptors Drd1 or Drd2 play different roles in social dominance behavior. Drd1-expressing neurons in the mPFC of social dominants showed greater excitatory synaptic transmission than did those in social subordinates. Higher mEPSC amplitudes and AMPA/NMDA ratios and lower rectification indices were observed in the mPFC of social dominants. In addition, the chemogenetic inhibition of prefrontal Drd1-expressing neurons in social dominants resulted in a reduction in social rank. However, the intrinsic excitability of prefrontal Drd2-expressing neurons was greater in social subordinates than in dominants, while there was no difference in the excitatory synaptic transmission of prefrontal Drd2-expressing neurons between social dominants and subordinates. Although chemogenetic inhibition of prefrontal Drd2-expressing neurons in social subordinates did not affect social hierarchy, simultaneous manipulation of prefrontal Drd1-expressing neurons to induce LTD in social dominants and prefrontal Drd2-expressing neurons to reduce neuronal activity in social subordinates switched the dominance-subordinate relationship (Fig. 3)^64^.

mPFC neurons send their axonal projections to various brain regions, and diverse mPFC projections may affect different aspects of social dominance behaviors. Biro et al. reported that optogenetic activation of the mPFC neuronal population projecting to the mediobasal hypothalamus (MBH) increases bite counts in resident-intruder conflicts, whereas optogenetic activation of mPFC projections in the lateral hypothalamus (LH) results in abnormal attacks. These findings show that mPFC projections to two different hypothalamic regions regulate social hierarchy by affecting distinct aggressive behaviors^65^. Another study revealed that the mPFC-LH circuit is involved in social dominance behavior. During the food competition test, the activity of mPFC-LH neurons was greater than that of mPFC neurons projecting to the basolateral amygdala during reward delivery in win trials. In addition, optogenetic activation of the mPFC-LH circuit increased reward consumption during the food competition test (Fig. 3)^61^. However, a recent study showed that mPFC-LH projections were not activated in the tube test. However, the mPFC-NAc and mPFC-VTA projections were highly activated in the winner and loser trials, respectively. This study also reported that effortful behaviors to win (i.e., push) or not to lose (i.e., resistance) increased mPFC-NAc projection activity. In contrast, retreat behavior increased mPFC-VTA projection activity. Optogenetic activation of mPFC-NAc (or mPFC-VTA) projections increased (or decreased) social ranks (Fig. 3)^34^. Similarly, another group recently reported that neurons in the ventromedial prefrontal cortex (vmPFC) that project to the NAc (vmPFC-NAc) of social subordinates show increased activity when pushing social dominants. However, when social dominants push subordinates, the activity of vmPFC-NAc neurons remains unchanged. In addition, the chemogenetic inhibition of vmPFC-NAc neurons in social subordinates but not dominants resulted in a reduction in pushing behaviors during the tube test^66^. In summarizing the roles of prefrontal projections associated with social dominance behaviors based on these previous studies, it is evident that mPFC-LH projections are associated with aggressive behavior during social interactions and success in food competition tests. Conversely, mPFC-NAc projections are activated during attempts to win in the tube test, while mPFC-VTA projections are involved in behaviors associated with losing.

Transcriptomic analyses of the mPFC revealed candidate genes related to social dominance behavior. Microarray analysis by Pallé et al. revealed 297 differentially expressed genes (DEGs) in the mPFC between social dominants and subordinates. These genes were associated with the cell cycle, proliferation, cell death, DNA binding and transcription, RNA binding, catalytic activity, actin and intermediate filament regulation, peptidase activity, and ATP binding. In addition, genes encoding olfactory and vomeronasal receptors were identified as DEGs: *Vmn1r232*, which is highly expressed in social dominants, and *Olfr131*, which is highly expressed in social subordinates^67^. Another study reported several long noncoding RNAs (lncRNAs), a vast and varied family of RNA transcripts with a length of more than 200 nucleotides for epigenetic control, in the mPFC that were differentially expressed between social dominants and subordinates via lncRNA microarray analysis. In particular, the levels of AK013786, antisense long noncoding RNA of synapsin II (AtLAS), and synapsin 2b (Syn2b) were significantly greater in social subordinates than in dominants. Knockdown of AtLAS or Syn2b in the mPFC resulted in elevated excitatory synaptic transmission and social ranks, whereas overexpression of these two genes in the mPFC decreased both excitatory synaptic transmission and social ranks (Fig. 2)^68^. A recent study reported mPFC projection-specific social dominance-related DEGs. In this study, single-cell RNA sequencing was performed using mPFC tissues from social dominants and subordinates, and mPFC-NAc or mPFC-VTA neurons were identified by the expression of previously reported projection-specific marker genes^69^. Transcriptomic analysis identified a social dominance-related DEG candidate, *Pou3f1*, in mPFC-VTA neurons. This gene was more highly expressed in social subordinates than in dominants, and this finding was validated via reverse transcription-quantitative polymerase chain reaction and fluorescence in situ hybridization experiments. This study also showed that the knockdown of *Pou3f1* in mPFC-VTA neurons of social subordinates results in an increase in social rank, whereas the overexpression of *Pou3f1* in mPFC-VTA neurons of social dominants reduces social rank^34^.

Other molecular mechanisms in the mPFC that are involved in social dominance behavior have been reported through various approaches other than transcriptomic analysis. Park et al. reported that chronic restraint stress (CRS) decreases social dominance by reducing the phosphorylation of Ser818 and Ser831 of the GluA1 subunit of a-amino-3-hydroxy-5-methyl-4-isoxazolepropionic acid receptors (AMPARs), which regulates AMPAR trafficking during synaptic plasticity, in the mPFC. In addition, the authors confirmed that these two phosphorylation sites are highly correlated with social winnings from the tube test (Fig. 2)^70^. Zhang et al. showed that postnatal forebrain excitatory neuron-specific deletion of cytoplasmic fragile X mental retardation 1 (FMR1)-interacting protein 2 (CYFIP2) results in increased filamentous actin (F-actin) levels and abnormal dendritic spines in L5 neurons of the mPFC. In addition, the mutant animal exhibited greater intrinsic excitability in mPFC neurons, resulting in higher social status^71^. Another recent study reported that specific genes in mPFC astrocytes are involved in social dominance behavior. Mice with astrocyte-specific deletion of cAMP response element-binding (CREB)-regulated transcription coactivator 3 (CRTC3) exhibited a lower social status with reduced functional connectivity between the mPFC and parietal cortex. The authors also found that astrocytic CRTC3 and CREB directly regulate amphiregulin secretion, resulting in an increase in social rank^72^. The epigenetic regulator histone deacetylase 2 (HDAC2), which is an enzyme that removes acetyl groups from histone proteins to tighten chromatin structure and inhibit gene expression, regulates social dominance. The gut dysbiosis induced by antibiotic treatment increased HDAC2 in the mPFC and decreased social dominance. Conversely, the knockdown of HDAC2 in the mPFC resulted in an elevation of social rank^73^.

### Nucleus accumbens

The NAc is part of the brain’s reward system and plays a central role in pleasure, motivation, and reinforcement learning. Approximately 95% of neurons in the NAc are GABAergic medium spiny neurons (MSNs) that express either Drd1 or Drd2. The NAc is divided into two subregions: the core, which is the inner structure of the NAc, and the shell, which is the outer region. This brain region is interconnected with the mPFC, amygdala, and VTA to coordinate responses to rewarding stimuli. Dysfunction in the NAc has been implicated in many neuropsychiatric disorders, such as addiction, depression, and schizophrenia. In addition, the NAc is involved in various aspects of social behavior, including social dominance.

Several studies have reported that activity in the NAc regulates social dominance behavior. Hollis et al. reported that social competition in rats activates the PFC as well as the NAc, especially Drd1-expressing MSNs (D1-MSNs) in this brain region, and inhibition of the NAc by injecting muscimol, a potent and selective agonist of GABA_A_ receptors, reduces social dominance (Fig. 3)^74^. Recent research has shown that subregions and cell types in the NAc differentially regulate social dominance behavior. Shan et al. found that injecting N-methyl-D-aspartic acid (NMDA) into the NAc core or shell results in lesions and a decrease in social rank (Fig. 3)^75^. These results are consistent with a recent finding reported by Choi et al., revealing a reduction in social dominance when inhibiting the mPFC-NAc circuit^34^. The authors also found that social subordinates exhibit greater mEPSC amplitude in D1-MSNs of the NAc shell than do dominants and that chemogenetic activation or inhibition of D1-MSNs in the NAc shell decreases or increases social hierarchy, respectively. In contrast, social dominants exhibited greater mEPSC amplitudes in D2-MSNs of the NAc core than did subordinates, and chemogenetic activation or inhibition of D2-MSNs in the NAc core increased or decreased social hierarchy, respectively (Fig. 3)^75^. This finding implies that D1-MSNs in the NAc shell encode social loss and subordination, whereas D2-MSNs in the NAc core encode social winning and dominance.

Both activity and energy metabolism in the NAc are involved in social dominance behavior. Hollis et al. reported that regulating mitochondrial function in the NAc changes social dominance. The inhibition of mitochondrial complexes I and II in the NAc induced social subordinates, whereas enhancing NAc mitochondrial function increased social dominance^74^. Another study showed that the metabolic profile in the NAc is related to social hierarchy in mice. ^1^H-nuclear magnetic resonance (^1^H-NMR) spectroscopy revealed that social dominants exhibit greater levels of energy-related metabolites such as Glx (glutamate + glutamine), total creatine (creatine + phosphocreatine), and taurine in the NAc than subordinates^76^. Recently, a molecule that affects social hierarchy through regulating mitochondrial function in the NAc was discovered. Mitofusin 2 (Mfn2), a mitochondrial GTPase that regulates various mitochondrial functions (e.g., respiration, volume regulation, and interactions with the endoplasmic reticulum), was shown to be expressed at diminished levels in the NAc of highly anxious animals that demonstrated depressive-like behaviors. These animals exhibited altered synaptic structure and function in the NAc. Overexpression of Mfn2 in the NAc of highly anxious animals mitigated all of these behavioral, mitochondrial, and synaptic abnormalities^77^. Intriguingly, the overexpression of Mfn2 in the NAc increases social dominance, whereas the knockdown of this gene in D2-MSNs but not D1-MSNs in the NAc induces social subordinates by reducing mitochondrial function and neuronal excitability^78^.

Several molecules in the NAc have been reported to be involved in social dominance behaviors. Aleyasin et al. reported that the expression of ΔFosB, a transcription factor known to regulate motivated behaviors and suggested to be a molecular switch for addiction, in the NAc is greater in aggressive mice, and overexpression of this gene in the NAc increases aggression and social dominance^79^. Another study showed that the expression of neuroligin-2 (Nlgn-2), a synaptic adhesion molecule that regulates synaptic structure and function, in the NAc is decreased by chronic stress and that the knockdown of this gene in D1-MSNs of the NAc reduces social dominance. However, the knockdown of Nlgn-2 in D2-MSNs of the NAc increases aggression and social dominance^80^. Various neurotransmitter or neuromodulator systems are also related to social dominance behavior. Androgen receptors were highly expressed in the NAc of social winners in territorial competition^81^. Social dominants had more oxytocin receptors in the NAc core than subordinates^82^, but the microinjection of oxytocin in the NAc shell reduced social ranks and effortful behaviors to win during the tube test^83^. Social subordinates demonstrating heightened anxious behaviors exhibited decreased expression levels of glucocorticoid receptor (GR) in the NAc compared to social dominants. Moreover, this investigation revealed that not only virally induced knockdown of GR expression in the NAc but also GR deletion in dopaminoceptive neurons (i.e., neurons expressing the dopamine Drd1 receptor) leads to heightened social dominance^84^. Recently, it was reported that the expression level of the cannabinoid 1 receptor in the NAc is greater in social subordinates than in dominants^85^.

### Ventral tegmental area

The VTA is located in the midbrain and serves as a hub of the mesocorticolimbic dopamine (DA) system, which plays a significant role in reward, motivation, and addiction behaviors. The two major projections of VTA DA neurons are the mesocortical and mesolimbic pathways, which correspond to the mPFC and NAc, respectively. Subpopulations of VTA glutamatergic or GABAergic neurons either regulate the surrounding VTA DA neurons or project to other brain regions. The VTA and its circuits are also involved in various aspects of social behaviors, including social hierarchy.

Inhibition of the VTA has been shown to increase social hierarchy. For example, van der Kooij et al. reported that the inhibition of the VTA via the microinfusion of diazepam, a positive allosteric modulator of GABA_A_ receptors, or muscimol increases social dominance (Fig. 3)^86,87^. These results are consistent with a recent finding reported by Choi et al., who demonstrated an increase in social dominance when inhibiting the mPFC-VTA circuit^34^. However, the authors explained that diazepam or muscimol acts on VTA GABAergic neurons rather than inhibiting the entire VTA. This action disinhibited VTA DA neurons, leading to an increase in DA release in the NAc, which, in turn, served as the mechanism that enhances social dominance^86–88^. Several studies have shown that the activation of VTA DA neurons increases social hierarchy. Optogenetic activation of VTA DA neurons increased agonistic and aggressive behaviors (Fig. 3)^89,90^ and decreased the latency to reward while increasing the reward obtained during the reward competition test, indicating an increase in social rank^41^. In contrast, Battivelli et al. reported that social dominants have lower VTA DA neuronal activity than subordinates and that the chemogenetic inhibition of VTA DA neurons increases social rank (Fig. 3)^91^.

Notably, DA release from the VTA to the NAc regulates social dominance behavior. Disinhibition of VTA DA neurons resulted in an increase in DA levels in the NAc, activating D1-MSNs in the NAc and elevating social hierarchy. In addition, the enhancing effect of diazepam on social dominance was reversed when diazepam was administered to the VTA, and a D1 receptor antagonist was administered to the NAc^85^. However, another study revealed that VTA DA neurons projecting to the lateral septum (LS) but not to the NAc regulate social dominance. Optogenetic activation of DAergic projections from the VTA to the LS but not to the NAc increased agonistic and aggressive behaviors. In addition, this effect disappeared when sulpiride, a D2 receptor antagonist, was locally infused into the LS^90^. These results suggest that DA release from the VTA to the NAc regulates social dominance behavior through the activation of Drd1 in the NAc, whereas DA release from the VTA to the LS controls aggressive behavior through the activation of Drd2 in the LS.

Multiple molecules in the VTA are related to social hierarchy. In both the NAc and the VTA, androgen receptors were highly expressed in social winners in territorial competition^81^. A transcriptomic analysis revealed 207 DEGs in the VTA, of which 89 (43%) exhibited reduced expression in social subordinates. These genes are related to DA synthesis and release, axon guidance, and drug addiction^92^.

## Clinical implications of circuit mechanisms related to social dominance

We investigated the neural mechanisms underlying social dominance behavior, focusing on the mPFC, NAc, and VTA, which are key components of the mesocorticolimbic and corticostriatal circuits. These brain regions are closely linked to both social dominance behavior and a broad spectrum of other behaviors and disorders.

The mPFC plays a pivotal role in various cognitive and emotional processes, and its dysfunction is associated with several neuropsychiatric disorders, including depression, anxiety disorders, posttraumatic stress disorder (PTSD), and schizophrenia. For example, hypoactivity or structural abnormalities in the mPFC are consistently implicated in depression^93^, while alterations in connectivity with certain brain regions (e.g., the amygdala) are typically observed in anxiety disorders^94^. Moreover, deficits in fear extinction and emotional regulation in the mPFC are linked to PTSD and impaired cognitive and social functioning in schizophrenia^95,96^.

The mesolimbic system, which mainly comprises the VTA and NAc, is critical in reward processing, motivation, and reinforcement learning. Dysregulation within this system is clinically relevant in various disorders, including addiction, depression, anxiety disorders, and mood disorders. For example, dysregulated dopamine signaling from the VTA to the NAc is a hallmark of addiction^97^, while altered reward processing in this system contributes to anhedonia and reduced motivation in depression and anxiety disorders^98^. Furthermore, dysfunctional mesolimbic activity is implicated in the pathophysiology of mood disorders such as bipolar disorder^99^.

The corticostriatal circuitry, including the mPFC-NAc projection, is essential for cognitive and motor functions, and its dysfunction is implicated in disorders such as obsessive-compulsive disorder (OCD), depression, anxiety disorders, and schizophrenia. Altered connectivity within this circuit is observed in OCD, suggesting its involvement in the disorder’s pathophysiology^100^. Similarly, abnormalities in circuit connectivity are linked to mood and anxiety disorders^101^. Additionally, aberrant dopamine signaling in the striatum within this circuit contributes to the pathophysiology of schizophrenia^102^.

## Behavioral correlates of social dominance

### Prosocial behaviors

Is there a correlation between social dominance and other social behaviors? As previously summarized, the mesocorticolimbic system is closely involved not only in social dominance behavior but also in sociability^9–11,21–29^. This involvement implies that sociability varies depending on social hierarchy. Šabanović et al. reported that social dominants interact much longer with an aggressor mouse before chronic social defeat stress (CSDS), whereas this difference disappears after CSDS^103^. In addition, Kunkel and Wang showed that socially dominant animals exhibit increased sociability. Socially top-ranked mice spent more time interacting with stranger mice than socially bottom-ranked mice during the three-chamber social interaction test^104^. These results suggest that socially dominant animals have greater social motivation.

Animals have the capacity for empathy. For instance, some previous studies revealed that when a mouse is subjected to electric foot shocks, an observing mouse exhibits fear responses. The intensity of observational fear response correlated with the familiarity between the mice, specifically how long they had lived together^105,106^. Is there a difference in empathy based on social hierarchy? Park et al. revealed that social subordinates (e.g., rank-4 mice in cages where four mice live together) show a greater observational fear response than rank-1 mice when an intermediate mouse (e.g., rank 2 or rank 3) is subjected to electric foot shocks. Additionally, an intermediate mouse showed greater observational fear response when dominants received electric foot shocks^107^.

Do animals engage in altruistic behaviors, and is there a relationship between these altruistic behaviors and social hierarchy? Scheggia et al. developed a two-choice social decision-making task in which mice could choose whether to share a reward with their conspecifics. They also found that social dominants make more altruistic choices than subordinates^108^.

### Stress

Stress profoundly influences various behaviors, notably social dynamics. Is stress a factor in shaping social hierarchies? An influential investigation by Cordero and Sandi focused on this question, with their pioneering study revealing that stress amplifies the recognition memory associated with social hierarchy. Initially, when two male rats engage in interactions and compete for food, a social hierarchy emerges but typically dissipates within a week. However, if one rat experiences stress induced by an electric foot shock before the initial encounter, the established dominance hierarchy persists beyond a week, with the stressed rat assuming a subordinate position. Additionally, anisomycin, a protein synthesis inhibitor, disrupts the long-term retention of social hierarchy memory^44^.

What are the underlying molecular mechanisms driving the enhancement of social hierarchy memory via stress? Stress triggers an increase in the stress hormone corticosterone, and its receptor, GR, is recognized not only for its involvement in stress responses but also in social dominance behavior. Timmer and Sandi demonstrated that stress-induced enhancement of social hierarchy memory is mediated by corticosterone. Specifically, they observed that systemic administration of corticosterone before a social encounter does not immediately affect the social hierarchy but promotes a lasting memory of the social hierarchy when the corticosterone-treated rat assumes a dominant role rather than a subordinate one. Additionally, administering corticosterone after a social interaction leads to the long-term maintenance of the social hierarchy only when the socially subordinate rat in the pair is injected with corticosterone^109^. However, these effects were not observed when corticosterone was injected into specific brain regions associated with social hierarchy establishment, such as the basolateral amygdala, medial amygdala (MeA), LS, and NAc^110^. These findings suggest that the enhancement of social hierarchy memory by corticosterone is attributable to its actions within the brain rather than specific brain regions. Notably, not only corticosterone and GR but also the oxytocin receptor are implicated in social hierarchy memory. Animals experiencing prolonged social subordination due to stress exhibit reduced expression levels of oxytocin receptors in the MeA. Furthermore, administering an antagonist of this receptor into the MeA immediately after acquiring a subordinate status under nonstress conditions induces the long-term establishment of the subordinate status^111^. Overall, these results indicate that stress influences the formation and maintenance of social hierarchies through its effects on various brain regions, neurotransmitter systems, and neuromodulators.

### Depression and anxiety

Some previous studies have reported contrasting findings regarding whether depression- or anxiety-like phenotypes vary based on social hierarchy. Fan et al. revealed that there were no differences in immobility in the forced swim test (FST) or sucrose preference in the sucrose preference test (SPT) based on the social status of the mice^112^. However, another study showed that there is an inverse relationship between social hierarchy and depression- and anxiety-like behaviors in mice. Using a tube test with four mice in a single group to establish social ranks, the authors categorized ranks 1 and 2 as social dominants and ranks 3 and 4 as social subordinates. Subsequently, in the tail suspension test (TST), greater immobility was observed in social subordinates than in dominants. Additionally, in the elevated plus maze test, social dominants spent more time in the open arms than did subordinates^37^. Similarly, it was revealed that a high anxiety phenotype is strongly correlated with social subordination in rats^44,74,86,87,109–111^.

How does stress induce changes in social dominants and subordinates? Larrieu et al. reported that social dominants and subordinates exhibit distinct phenotypes after CSDS. Social dominant mice that underwent CSDS exhibited decreased social interaction with an aggressor mouse, whereas social subordinates exhibited a resilient phenotype after chronic stress^76^. However, LeClair et al. showed that social interaction with an aggressor mouse is reduced in male social subordinates but not social dominants after CSDS. In females, CSDS resulted in reduced social interaction with an aggressor mouse in both social dominants and subordinates, whereas vigilance was increased only in social subordinates after CSDS. Additionally, male and female subordinates are susceptible to chronic variable stress^36^. Interestingly, it was discovered that a history of prior stress regulates social hierarchy. After introducing a mouse that had previously experienced CSDS to a group of other mice and determining their social hierarchy, it was observed that their win ratio was relatively high^103^.

When socially dominant animals lost their social status, they exhibited depressive-like behaviors. Two previously dominant or subordinate mice from different cages were housed together, after which the tube test was performed. After CSDS, dominant mice that remained dominant had greater social interaction with an aggressor than dominant mice that became subordinate^36^. When socially subordinate mice were prevented from exiting the tube during the tube test, which forced social dominants to yield, the originally dominant animals lost their social status and showed depressive-like behaviors^112^.

### Worker-parasite relationship

The term “worker-parasite” was encapsulated within the conceptual framework delineated by Masur et al. in 1977^113^. However, this phenomenon was described as a “social problem,” as documented by H. Oldfield-Box in 1967, which served as the basis for creating an unbalanced workload model^114,115^. The worker-parasite relationship is explained by the different behaviors used to compensate, in which one of the rats continues to press the lever, while the other two consume the reward without working. Notably, these relationships occur regardless of sex^116^, but they exhibit some differences depending on social characteristics.

Previous studies have shown that individuals isolated from other individuals tend to become parasites; interpreted differently, more socially experienced individuals are likely to become workers^117^. In another report, the worker and parasite had similar aggressiveness before separation, but after separation, the worker (or parasite) took aggressive (or defensive) positions^118^. This finding suggests that aggression is a result, not a determinant, of social interaction in the rat. This result is consistent with those of several social hierarchy tests performed by Ahn et al.^119^. They revealed that the worker takes the dominant social rank under food reward conditions (e.g., feeder test and cylinder test), whereas it takes the subordinate social rank without any reward (e.g., tube competition test). Importantly, these characterizations were clearly inconsistent with perfect matches, as there was no food reward in the context of the general tube competition test. Although behavioral studies on dominance and food reward relationships have been performed^41–43,114,118,120^, the “worker-parasite relationship” cannot be explained by a simple, socially dominant hierarchy. The correlation between social hierarchy and the “worker-parasite” nexus seems to be determined by the structure of the test and the type of reward^121–124^.

## Conclusion and future directions

The tube test is a simple and straightforward behavioral test for investigating social hierarchy in rodents. However, one limitation of this test is its ability to assess social dominance only between pairs of animals. When dealing with multiple animals within a group, conducting multiple tube tests in a round-robin manner can establish social ranks, but it fails to capture interactions involving more than two animals, thus hindering the study of social rank formation. Additionally, in cases where stable social hierarchies do not emerge during tube tests, it remains ambiguous whether the group genuinely lacks a social hierarchy or if it simply goes undetected through this method.

Furthermore, the interpretation of the tube test as a territorial competition is uncertain, given that the tube test represents an artificial environment not reflective of the animals’ natural habitats. Thus, occupying this space may not necessarily confer advantages for survival. Nevertheless, despite its limitations, the tube test remains the predominant method for studying social dominance behavior in rodents due to the absence of superior alternatives.

To address the aforementioned limitations, it is imperative to develop additional behavioral tests for studying social dominance behavior and validating correlations. Furthermore, devising techniques for automatically analyzing various social competition behaviors, such as pushing and retreating, observed during the tube test is essential. Ultimately, there is a pressing need for research methodologies facilitating the observation and analysis of interactions and competition among multiple animals in their natural environments.

In this review, we have outlined the involvement of brain regions in social dominance behavior, with a focus on the mesocorticolimbic system. However, our understanding of the constituent areas, specific cell types, and their connections in regulating social hierarchy remains incomplete. Similarly, our knowledge of the genes within the mesocorticolimbic system that contribute to social hierarchy formation and their functional roles in this process is limited. Therefore, further research is warranted to elucidate these aspects comprehensively. Additionally, investigating whether brain regions beyond the mesocorticolimbic system influence social dominance behavior is crucial. This necessitates research exploring brain areas and neural circuits involved in social dominance behavior at the whole-brain level. For instance, the identification of neurons and their projections activated by social competition and dominance behaviors can be accomplished by detecting immediate early genes (e.g., c-Fos and Arc)^125,126^ or through the use of innovative tools such as the Calcium and Light-induced Gene Handling Toolkit (Cal-Light)^127,128^, the Fast Light Activity Regulated Expression (FLARE)^129^, or the Fast Light and Calcium-Regulated Expression (FLiCRe)^130,131^. Mapping of activated neurons and their projections throughout the entire brain can be achieved using conventional histological methods^34^, serial two-photon tomography^132–134^, or three-dimensional imaging techniques (e.g., light sheet microscopy) combined with tissue clearing^135–137^. Alternatively, neuroimaging techniques, such as manganese (Mn^2+^)-enhanced magnetic resonance imaging (MEMRI), provide a noninvasive approach to identifying brain regions and circuits involved in social hierarchy. In MEMRI, Mn^2+^ serves as a proxy for calcium (Ca^2+^) influx, as it can enter neurons through voltage-gated Ca^2+^ channels and accumulate in neurons in an activity-dependent manner. Consequently, MEMRI enables the visualization of neuronal activity history^138,139^, facilitating the delineation of activated brain regions and circuits in social dominants or subordinates.
